# Causes of endoscopic misdiagnosis of gastrointestinal cyst as solid lesion

**DOI:** 10.1186/s12876-022-02545-x

**Published:** 2023-01-11

**Authors:** Fei Gao, Huikai Li, Chen Du, Ke Han, Enqiang Linghu

**Affiliations:** Senior Department of Gastroenterology, the First Medical Center of PLA General Hospital, Beijing, China

**Keywords:** Gastrointestinal cyst, Gastrointestinal submucosal tumor, Endoscopic ultrasound

## Abstract

**Background:**

To explore the causes of endoscopic misdiagnosis of gastrointestinal cyst as solid lesion and the diagnostic value and limitations of EUS, guide clinicians to develop appropriate treatment strategies and improve the ability to identify SMT.

**Methods:**

We enrolled patients diagnosed with gastrointestinal SMT between January 2001 and December 2021 who underwent endoscopic resection with postoperative pathological diagnosis of cyst. Age, sex, maximum lesion diameter, judge the texture of lesion, origin and echo are potential factors affecting the diagnostic accuracy of cysts.

**Results:**

The diagnostic accuracy of EUS assessment 39.3% higher than that without EUS assessment (6.7%). The error rate was 60.7%, lower than that without EUS assessment (93.3%), suggesting that preoperative EUS assessment improved the diagnostic accuracy of gastrointestinal cyst (Fisher's accurate test, *P* = 0.033). The diagnostic accuracy of “judge the texture of lesion” was higher than that of no touch (*P* = 0.031). When the lesion size increased by 1 cm, the diagnostic accuracy decreased by about 21%. Hypoechoic lesions were less likely to be diagnosed correctly than anechoic lesions (*P* = 0.003).

**Conclusions:**

The main cause of misdiagnosing gastrointestinal cyst as solid lesion is that no EUS assessment was performed before endoscopic resection or anechoic lesion was judged as hypoechoic lesion by preoperative EUS assessment.

## Background

Due to a growing awareness of physical examination, the improvement of endoscope and endoscopic ultrasound (EUS) and the development of related equipment and techniques, the detection rate of gastrointestinal submucosal tumor (SMT) has been greatly increased. Super minimally invasive surgery (SMIS) [1] for gastrointestinal SMT, with the advantages of maintaining the integrity of gastrointestinal anatomical structure, less trauma, fewer complications, and no postoperative impact on patients' quality of life, is currently the best treatment for SMT. As it is difficult to diagnose the types of SMT by endoscopy alone, EUS is employed to further clarify the types of SMT and provide guidance for the treatment by probing the size, originating layer, echo level, and internal echo pattern [2]. Endoscopic resection is suitable for lesions without lymph node metastasis or with very low risk of lymph node metastasis, as well as lesions that can be completely removed by endoscopic technique with low risk of residual and recurrence [3].

As a type of SMT, cysts have no obvious morphological specificity, are anechoic or hypoechoic. Without systematic assessment, it is difficult to distinguish cysts from other SMT. If the type of SMT are not diagnosed before surgery, the cyst that does not need to be resected may be misdiagnosed as solid lesions. However, in clinical practice, many cysts are directly removed by endoscopic or surgical resection without being identified. Although SMIS can retain the original anatomical structure, it still takes time and money, resulting in unnecessary losses. There are plenty of studies on the indications of SMT, but none specific to cyst. The purpose of this study is to explore the causes of endoscopic misdiagnosis of gastrointestinal cyst as solid lesion and the diagnostic value and limitations of EUS, guide clinicians to develop appropriate treatment strategies and improve the ability to identify SMT.

## Methods

We enrolled patients in the First Center of PLA General Hospital from January 2001 to December 2021. Inclusion criteria are as follows: 1. Endoscopic diagnosis was gastrointestinal SMT; 2. The SMT was resected by endoscope (SMIS). Exclusion criteria are as follows: 1. The specimen was not sent for pathology after resection or deficiency; 2. Postoperative pathological diagnosis was not cyst. 524 patients diagnosed SMT underwent endoscopic resection, and 481 cases were excluded. 43 cases performed by 18 experienced endoscopist with senior title were enrolled. The endoscopic workstation collected the general information of the patients and their pathological characteristics and endoscopic diagnosis during preoperative evaluation by white light endoscopy and EUS, as well as postoperative pathological diagnosis.

### Equipment and instruments

Endoscope: Olympus GIF-H260/GIF-H290. Probe EUS: Olympus Ultrasonic Probe Set MH-247, Olympus Ultrasonic Probe UM-2R/UM-3R, Olympus Probe Driving Unit MAJ-935, FUJINON SONOPROBE SP-701. EUS: Olympus GF-UCT260. Ultrasonic diagnostic system: ALOKA Prosound F75.

### Definition

General procedures of preoperative evaluation: Blood routine, blood biochemistry, coagulation function and infectious diseases were tested, and electrocardiogram and chest X-ray /CT conducted after admission. White light endoscopy was performed to observe the lesion site and surface size. Whether to touch the lesion with biopsy forceps to judge its texture was determined according to the operator's habit. Cysts are usually soft and easily deformed when touched with biopsy forceps. It was up to the operator to decide whether to use EUS to assess the origin, sectional size, and echo of the lesion.

Diagnosis of digestive tract cyst: 1. Combination of fluid outflow in endoscopic incision of tumor and pathological diagnosis of biopsy of the wall of tumor; 2. Pathological diagnosis of postoperative specimen was cyst.

**Fig. 1:**
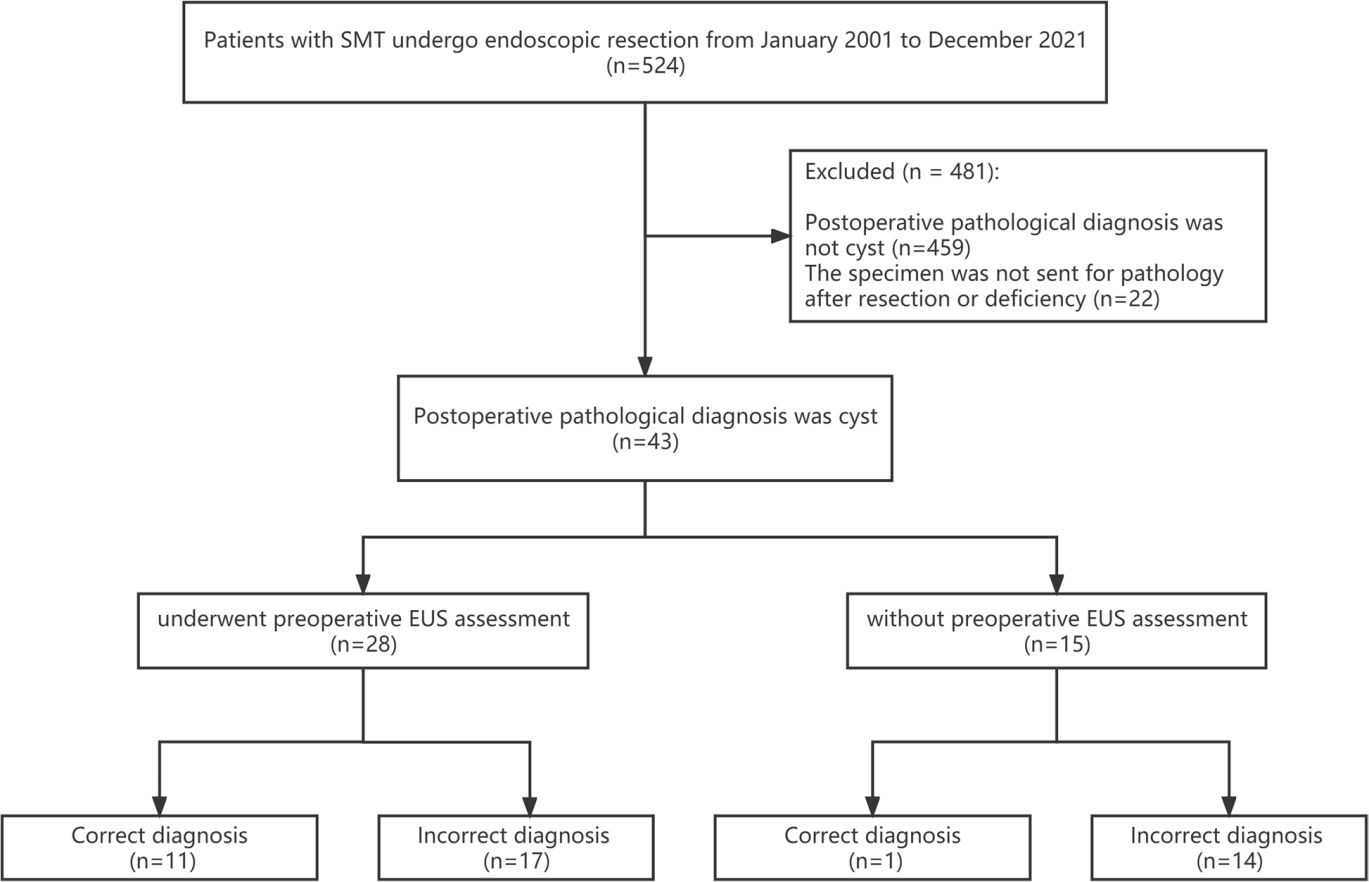
Study flowchart

Correct diagnosis & incorrect diagnosis: If the preoperative endoscopic diagnosis is the same as the postoperative pathological diagnosis, the diagnosis is correct; otherwise, it is incorrect. The study flowchart is shown in Fig. 1

### Statistical analysis

Descriptive statistics were presented as medians [interquartile range (IQR)] for non-normally distributed continuous data and frequencies (percentage) for categorical data.The differences between the medians and proportions of the two groups were compared by analysis of Mann–Whitney U test and chi-square test or Fisher's exact test.Univariate logistic regression analysis was conducted to determine whether gender, age, lesion size and texture judged by touch, lesion origin and lesion echo had any influence on the diagnostic accuracy. A two-tailed *P*-value of less than 0.05 was considered statistically significant. Analyses were completed using SPSS version 25.0 (IBM Corp, Armonk, NY, USA).

## Results

A total of 43 cases were collected, including 31 males (72.1%) and 12 females (27.9%) with an age range of 19–76 years (mean 51 years). Lesions size were 0.3–5.0 cm, with a median size of 0.7 cm. The lesions were located in esophagus in 39 cases, stomach in 2 cases, duodenum in 1 case and colorectum in 1 case. Of the 43 cases, 28 cases underwent preoperative EUS assessment and 15 cases did not. In total 14 cases in this study underwent endoscopic resection with unknown preoperative diagnosis, and these 14 cases were classified as misdiagnosis, because the cysts did not need to be resected.

The results of preoperative endoscopic and EUS assessment and postoperative pathological diagnosis were compared. Without EUS assessment, the diagnosis was correct in 1 case and wrong in 14 cases. After EUS assessment, 11 cases were diagnosed correctly and 17 cases were diagnosed incorrectly. During preoperative endoscopy, biopsy forceps were used to touch the lesion to judge its texture in 6 cases. Echo pattern was assessed by EUS: 13 cases were anechoic, 14 cases were hypoechoic, and 1 case was hyperechoic. The origin of lesions was assessed by EUS: mucosal/muscularis mucosa in 16 cases, submucosal in 10 cases, and undescribed in 2 cases (Table 1).

**Table 1:** Baseline characteristics of patients and gastrointestinal cyst

	Correct diagnosis(*n* = 12)	Incorrect diagnosis(*n* = 31)	Total	*P* Value
Age, y	49.00 (36.00–56.00)	54.00 (44.00–63.00)	52.00 (42.50–62.00)	0.316
Sex				0.307
Male	10 (83.33%)	21 (67.74%)	31 (72.09%)	
Female	2 (16.67%)	10 (32.26%)	12 (27.91%)	
Maximum lesion diameter, cm	1.25 (0.65–1.62)	0.70 (0.50–1.00)	0.70 (0.50–1.10)	0.071
Location of lesion				0.29
Esophagus	11 (91.67%)	28 (90.32%)	39 (90.70%)	
Stomach	0 (0.00%)	2 (6.45%)	2 (4.65%)	
Duodenum	1 (8.33%)	0 (0.00%)	1 (2.33%)	
Colorectum	0 (0.00%)	1 (3.23%)	1 (2.33%)	
Judge the texture of lesion				0.042
Yes	4 (33.33%)	2 (6.45%)	6 (13.95%)	
No	8 (66.67%)	29 (93.55%)	37 (86.05%)	
Preoperative EUS assessment				0.023
Yes	11 (91.67%)	17 (54.84%)	28 (65.12%)	
No	1 (8.33%)	14 (45.16%)	15 (34.88%)	

The chi-square test indicated that whether EUS assessment was performed before SMIS had different diagnostic accuracy, with the diagnostic accuracy of EUS assessment 39.3% higher than that without EUS assessment (6.7%). The error rate was 60.7%, lower than that without EUS assessment (93.3%), suggesting that preoperative EUS assessment improved the diagnostic accuracy of gastrointestinal cyst (Fisher's accurate test, *P* = 0.033). Univariate logistic regression analysis showed that the diagnostic accuracy of “judge the texture of lesion” (with the biopsy forceps) was higher than that of no touch (*P* = 0.031) (Table 2).

**Table 2:** Baseline characteristics of patients and gastrointestinal cyst (with preoperative EUS assessment)

	Correct diagnosis(*n* = 11)	Incorrect diagnosis(*n* = 17)	Total	*P* Value
Age, y	49.00 (35.00–53.50)	55.00 (47.00–64.00)	53.00 (44.50–59.25)	0.109
Sex				0.249
Male	9 (81.82%)	10 (58.82%)	19 (67.86%)	
Female	2 (18.18%)	7 (41.18%)	9 (32.14%)	
Maximum lesion diameter, cm	1.50 (0.75–1.75)	0.70 (0.50–1.00)	0.80 (0.50–1.20)	0.035
Location of lesion				0.393
Esophagus	10 (90.91%)	17 (100.00%)	27 (96.43%)	
Duodenum	1 (9.09%)	0 (0.00%)	1 (3.57%)	
Judge the texture of lesion				0.062
Yes	4 (36.36%)	1 (5.88%)	5 (17.86%)	
No	7 (63.64%)	16 (94.12%)	23 (82.14%)	
Origin				0.151
Mucosal/muscularis mucosa	4 (36.36%)	12 (70.59%)	16 (57.14%)	
Submucosa	6 (54.55%)	4 (23.53%)	10 (35.71%)	
Undescribed	1 (9.09%)	1 (5.88%)	2 (7.14%)	
Echo				< 0.001
Anechoic	10 (90.91%)	3 (17.65%)	13 (46.43%)	
Hypoechoic	1 (9.09%)	13 (76.47%)	14 (50.00%)	
Hyperechoic	0 (0.00%)	1 (5.88%)	1 (3.57%)	

When the lesion size increased by 1 cm, the diagnostic accuracy decreased by about 21%. There was no statistically significant difference in the probability of correct diagnosis between submucosa (*P* = 0.135) and undescribed (*P* = 0.472) compared with mucosal/mucosal muscle layer. Hypoechoic lesions were less likely to be diagnosed correctly than anechoic lesions (*P* = 0.003) (Table 3).

**Table 3:** Factors affecting the diagnostic accuracy of cysts

Factor	OR	95%CI	*P* Value
All patients (*n* = 43)			
Sex			0.316
Male	1		
Female	2.381	0.437–12.964	
Age	1.030	0.979–1.084	0.250
Maximum lesion diameter	0.212	0.048–0.939	0.041
Judge the texture of lesion			0.038
No	1		
Yes	7.250	1.118–47.000	
Patients with preoperative EUS assessment (n = 28)			
Origin			0.212
Mucosal/muscularis mucosa	1		
Submucosa	4.500	0.824–24.568	
Undescribed	3.000	0.150–59.890	
Echo			0.009
Anechoic	1		
Hypoechoic	0.023	0.002–0.257	

Take the following two cases as examples, whether the echo of SMT is hypoechoic or anechoic, the lesion may be a cyst.

### Case 1

**Fig. 2:**
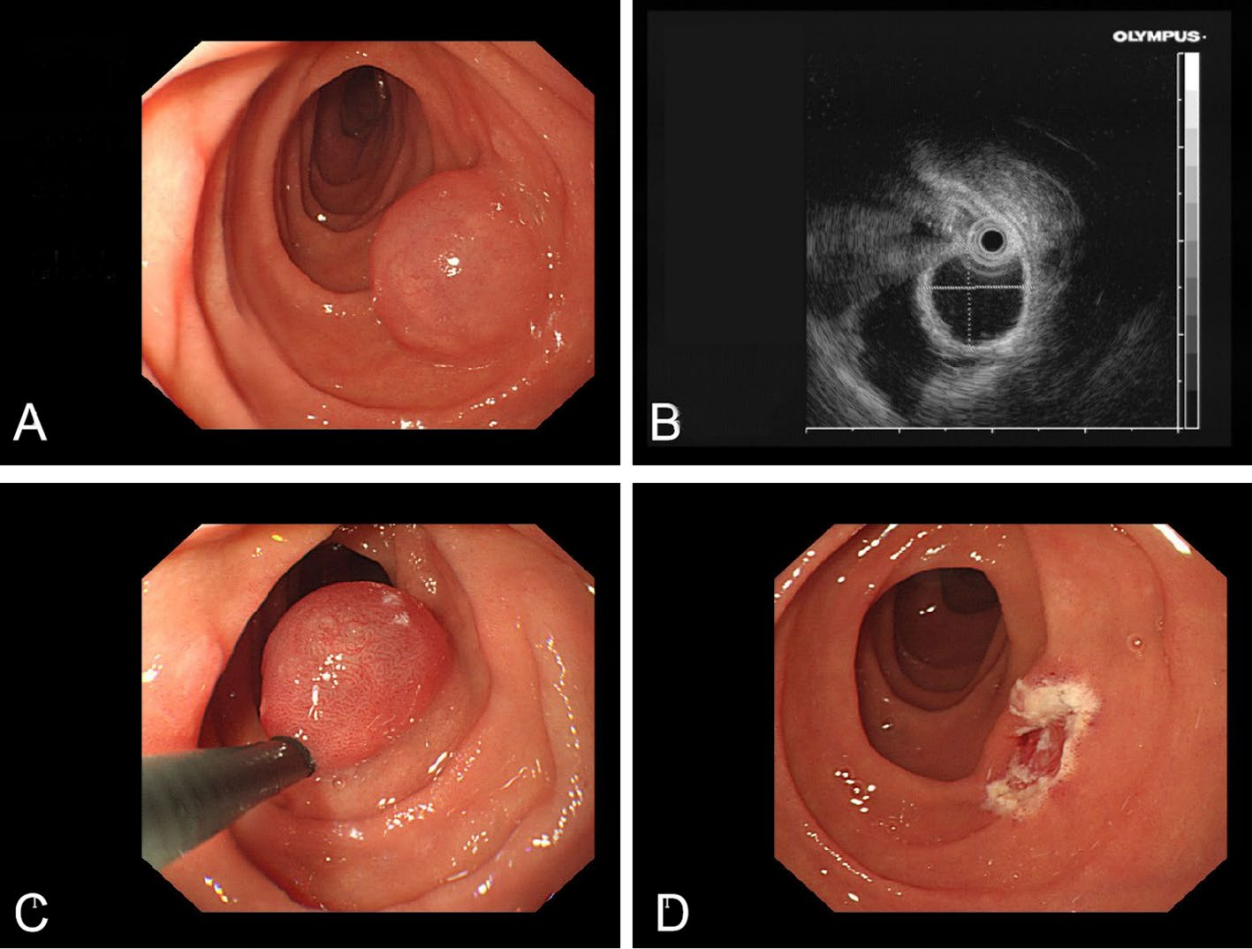
**A** spherical SMT was observed in duodenum; **B** EUS showed that the lesion had an anechoic cystic structure; **C** Most of the cystic wall was removed by electrocoagulation with snare; **D** The capillaries of residual basal cystic wall were clear

In an elderly male, a spherical SMT about 1.5 cm in diameter was observed in the descending part of duodenum. The surface was smooth and translucent. EUS showed that the lesion originating from submucosa had an anechoic cystic structure with a smooth wall, and musculus propria was intact. Presumptive diagnose is cyst. Most of the cyst wall (about 3/4) were removed by electrocoagulation with a snare, and part of basal capsule wall were left, and the capillaries in the wall were clear (Fig. 2). The specimen was sent for pathological examination and was diagnosed as cyst.

### Case 2

**Fig. 3:**
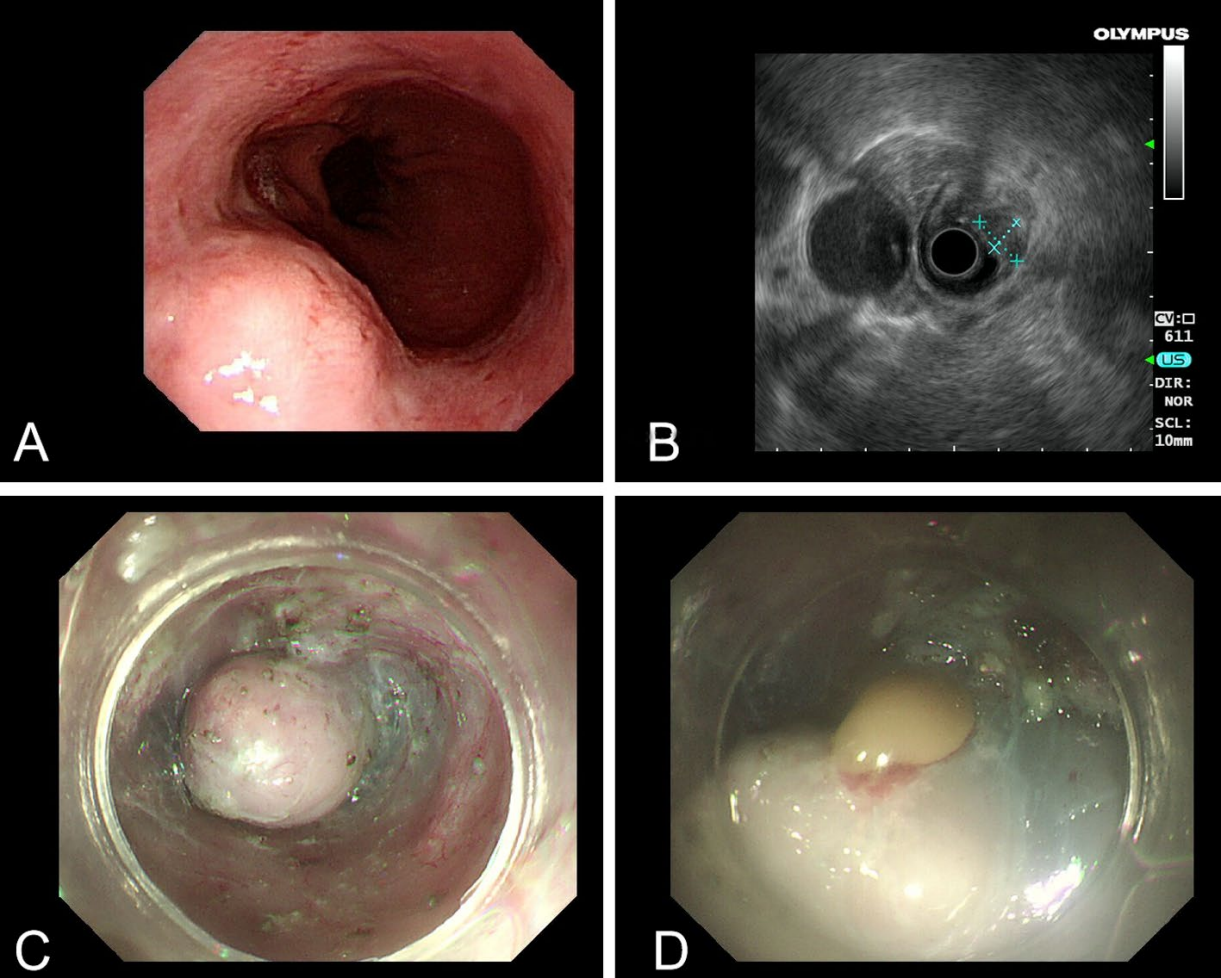
**A** SMT was observed in the esophagus 38 cm from the incisors; **B** The lesion was mainly hypoechoic in EUS; **C** A tunnel was established to fully expose the lesions during endoscopic resection; **D** Viscous yellow fluid was released from the lesion

A SMT approximately 2.0 cm × 1.0 cm in size located in esophagus 38 cm from the incisors was observed. The lesions in EUS were mainly hypoechoic, and some were mixed with iso-echoic. The lesion originated from the submucosa and the superficial layer of muscularis propria, with clear boundary and regular morphology, and there was no blood flow signal under Doppler. The cross-section size was about 7.7 mm × 12.3 mm. Presumptive diagnose is not cyst. During endoscopic resection of esophageal SMT, a small amount of viscous yellow fluid was seen flowing from the lesion (Fig. 3).

## Discussion

This study retrospectively analyzed the data of the patients diagnosed with gastrointestinal SMT in our hospital from 2001 to 2021 who received endoscopic resection and the SMTs were pathologically diagnosed as cysts. After data analysis, EUS assessment before surgery improved the diagnostic accuracy of cysts. For the origin of lesion, there was no significant difference in the probability of correct diagnosis in submucosa and undescribed compared with mucosal/ muscularis mucosa. The probability of correct diagnosis of hypoechoic lesions was reduced compared with anechoic lesions. In addition, the accuracy of diagnosis was improved by touching the lesion with biopsy forceps to determine its texture, which is a novel, simple and effective method worth popularizing.

Patients with SMT have no specific clinical manifestations and SMT is usually found during screening endoscopy. However, endoscopy can only observe the surface morphology of the SMT and the presence of ulcer, but cannot diagnose the nature and origin of the lesions. It is especially difficult to distinguish SMT from extralimentary compression lesions. Although SMT can be further examined by EUS to clarify the origin and echo of lesions and help clinicians to develop treatment strategies, EUS has certain limitations. The cross sections of lesion are different in EUS, resulting in the constant change of origin and size. The echo pattern may be inconsistent due to the different resolution of EUS devices or the experience of endoscopist. Non-standard EUS procedures can easily lead to misdiagnosis and thus affect treatment strategies. The diagnosis of SMT can be combined with EUS and other imaging examinations, such as CT or MR, but the gold standard of diagnosis is the pathological diagnosis, with the specimen obtained by EUS-guided fine-needle aspiration (EUS-FNA) or after endoscopic resection [4, 5].

Gastrointestinal cysts are usually characterized by smooth and regular SMT under endoscopy. Those with shallow origins may appear translucent and are soft and easy to be compressed when touched by biopsy forceps. By EUS examination, cysts can originate from any layer or even outside the wall, and the lesions are anechoic, round or quasi-round. There are partitions or deposits in some cysts, with no blood flow signal in color Doppler. Fluid outflow from the cyst can be observed after destruction of the mucosal surface of the cyst during biopsy or diagnostic resection.

The most common solid lesions in SMT are leiomyoma and stromal tumor, and it has been reported that CE-EUS can play a certain role in differentiating them [6–8], because the blood flow signal distribution characteristics in lesions are different after angiography. Considering that there is no blood flow signal distribution under CE-EUS as there is no blood vessel in the fluid component of the cyst, CE-EUS is likely to be more accurate in identifying the cyst consolidation of SMT. However, studies have shown that CE-EUS differentiates the malignant risk prediction of subepithelial tumors and mesenchymal tumors, but the results still need to be confirmed by further studies [9].

This study has the following limitations. Firstly, this is a retrospective study with inherent bias. Secondly, this study lacks representativeness because it is a single-center study, the operators are all experienced endoscopist and the equipment used is in a leading position in China. Finally, there is no clear objective criteria for anechoic and hypoechoic lesions. In the follow-up study, we plan to develop specific evaluation criteria for echo based on EUS standard atlas method, so as to clearly distinguish anechoic and hyperechoic lesions, and further reduce the misdiagnosis rate of gastrointestinal cyst.

## Conclusions

The main cause of misdiagnosing gastrointestinal cyst as solid lesion is that no EUS assessment was performed before SMIS or anechoic lesion was judged as hypoechoic lesion by preoperative EUS assessment. During the diagnosis of SMT, after the surface morphology of the lesion is observed, the biopsy forceps can be used to gently touch the lesion to determine its texture. Then, EUS is used to carefully observe the origin and echo of lesions according to the standard spectrum method [10]. In case of "hypoechoic" lesions, vigilance should be raised and repeated scanning of lesions should be performed to make differential diagnosis.

## Data Availability

The datasets generated and/or analysed during the current study are not publicly available due [Our hospital is a military hospital, and some data are classified] but are available from the corresponding author on reasonable request.
